# Spatial and Temporal Distribution Characteristics of VOCs in Seoul Ambient Air and Identification of Potential Pollution Sources Using Principal Component Analysis

**DOI:** 10.3390/toxics14070554

**Published:** 2026-06-25

**Authors:** Ji-Yun Jung, Shin-Young Park, Ye-Jin Sim, Jong-Cheol Yoon, Hak-Myeong Lim, Kwang-Rae Kim, Seok-Ryul Oh, Yong-Suk Choi, Cheol-Min Lee

**Affiliations:** 1 Department of Chemical and Environmental Engineering, Seokyeong University, Seoul 02713, Republic of Korea; jju1049@skuniv.ac.kr (J.-Y.J.); tlsdud060900@skuniv.ac.kr (S.-Y.P.); yejins@skuniv.ac.kr (Y.-J.S.); 2 Seoul Metropolitan Government Research Institute of Public Health and Environment, Seoul 13818, Republic of Korea; yoonkjc@seoul.go.kr (J.-C.Y.); chayke1@seoul.go.kr (H.-M.L.); kimkr@seoul.go.kr (K.-R.K.); ifnwu@seoul.go.kr (S.-R.O.); hozer87@seoul.go.kr (Y.-S.C.)

**Keywords:** volatile organic compounds, urban air quality, spatial variations, seasonal variations, principal component analysis

## Abstract

This study analyzed the spatial distribution and seasonal variation characteristics of Volatile Organic Compounds (VOCs) at four sites (GS, GJ, BHS, and JN) representing different emission environments in Seoul and identified potential pollution sources using principal component analysis (PCA). The results showed that VOC concentrations were relatively high at the GS site, which is influenced by both industrial and traffic emissions, and at the JN site, characterized by heavy urban traffic, whereas the BHS site, representing a background area, exhibited the lowest concentrations, indicating clear spatial heterogeneity. Alkanes accounted for the largest proportion of VOCs at all sites, and low-molecular-weight alkanes as well as combustion-related compounds showed elevated concentrations during winter. In contrast, aromatic compounds exhibited site-specific seasonal patterns, with relatively higher concentrations observed during summer or autumn at some locations. The diurnal variation patterns displayed a bimodal distribution with concentration peaks during morning and evening rush hours, indicating the direct influence of traffic emissions. Furthermore, the T/B ratio and PCA results suggested that vehicle emissions and combustion sources were the dominant contributing factors (PC1) to ambient VOCs in Seoul, while non-road emission sources such as solvent use and industrial activities, characterized mainly by aromatic compounds, also contributed significantly (PC2). The findings of this study can serve as fundamental data for future quantitative source apportionment studies and the development of risk-based air quality management strategies for VOCs in Seoul.

## 1. Introduction

Volatile Organic Compounds (VOCs) are major precursors that play an important role in the formation of Ozone (O_3_) and Secondary Organic Aerosols (SOAs) through photochemical reactions in the atmosphere. VOCs react with nitrogen oxides (NO_x_) under sunlight to produce O_3_, which has been reported to contribute not only to the deterioration of urban air quality but also to adverse effects on human health [1,2]. In addition, some VOCs are classified as hazardous air pollutants due to their carcinogenic and toxic properties and may cause various health problems, including respiratory diseases and neurological effects [3]. Therefore, understanding the concentration characteristics, temporal variability, and source-related behavior of VOCs in the urban atmosphere is essential for effective air quality management. Although VOCs are associated with adverse health effects, the primary focus of this study is to characterize their spatial and temporal distribution patterns and potential emission sources.

Ambient VOC concentrations in urban areas are influenced by various anthropogenic emission sources, including traffic, fuel combustion, industrial activities, and solvent usage, as well as biogenic emissions. These sources exhibit substantial spatial and temporal variability depending on regional characteristics, meteorological conditions, and seasonal changes [4]. In particular, megacities such as Seoul, characterized by high population density, heavy traffic volume, and diverse emission sources, show pronounced spatial heterogeneity in VOC concentrations. Previous studies conducted in Seoul have reported that traffic emissions and solvent use are major sources of VOCs, while long-range transport from surrounding regions also significantly affects urban VOC levels [3]. Furthermore, VOCs consist of a complex mixture of numerous chemical species, making it important to analyze not only concentration levels but also interspecies relationships and compositional characteristics. Previous studies have shown that VOC concentrations exhibit significant seasonal and diurnal variations, with relatively higher concentrations commonly observed during spring, winter, and daytime periods [5]. These patterns are interpreted as the combined effects of anthropogenic emission activities, photochemical reactions, and atmospheric dispersion processes.

For source identification, both emission-based approaches using emission inventory data and receptor-oriented approaches based on measured concentration data have been widely applied [6]. Representative receptor models include Chemical Mass Balance (CMB), Principal Component Analysis (PCA), UNMIX, and Positive Matrix Factorization (PMF). Among these, the PMF model has been most widely used for VOC source apportionment because it can estimate emission sources without requiring predefined source profiles [7,8]. PMF has been effectively applied in numerous VOC-related studies to distinguish major emission sources such as traffic, industrial activities, and solvent use [9,10]. However, PMF results are highly dependent on the characteristics of the input data and atmospheric chemical transformation processes. In particular, uncertainties may arise in the interpretation of highly reactive compounds such as VOCs [11,12]. Therefore, a thorough understanding of the spatial and temporal distribution characteristics of VOCs, as well as the relationships among chemical species, is essential prior to conducting PMF analysis. In this context, PCA can serve as a useful exploratory tool prior to PMF analysis by identifying major patterns of covariance among VOC species, evaluating relationships between compounds, and screening potential source-related groupings. Such preliminary information may help interpret PMF factors and assess the suitability of VOC species included in receptor modeling [13].

However, previous VOC studies conducted in Seoul have primarily focused on short-term monitoring campaigns, individual monitoring sites, or source apportionment analyses [14]. Comprehensive evaluations of long-term spatial and temporal VOC characteristics across multiple monitoring environments remain limited. In particular, few studies have systematically compared VOC distribution patterns among traffic-influenced, mixed urban, industrially influenced, and background environments using long-term monitoring datasets. The novelty of this study lies in the use of a long-term, hourly resolved VOC monitoring dataset collected from four sites representing distinct emission environments in Seoul. Such comprehensive multi-site evaluations remain limited despite their importance for understanding urban VOC variability and supporting future source-apportionment studies.

Therefore, this study analyzed a long-term (2018–2024) VOC monitoring dataset collected at four sites representing different emission environments in Seoul. The objectives were to (1) characterize the spatial, seasonal, and diurnal variability of VOC concentrations, (2) evaluate inter-species relationships and source-related indicators, and (3) identify potential emission characteristics using PCA as a preliminary step toward future PMF-based source apportionment. Because the primary objective of this study was to investigate long-term spatial and temporal characteristics of VOCs and explore source-related patterns across multiple monitoring environments, PCA was employed as an exploratory multivariate technique rather than a quantitative receptor model. The findings are intended to provide fundamental information for future PMF-based source apportionment and risk assessment studies of VOCs in Seoul. However, direct health risk assessment was beyond the scope of the present study. Among the 56 VOC species measured at the measurement sites, 16 compounds with detection frequencies exceeding 50% were selected for detailed analysis to ensure statistical reliability and reduce uncertainty associated with infrequently detected species. These compounds represent major VOC groups commonly used in urban air quality and source characterization studies.

## 2. Materials and Methods

### 2.1. Measurement Sites

In this study, hourly VOC data collected from January 2018 to March 2024 were obtained from the Photochemical Assessment Monitoring Stations (PAMSs) operated by the Seoul Metropolitan Government Research Institute of Health and Environment (SIHE) at four sites in Seoul, South Korea: Gangseo (GS), Gwangjin (GJ), Bukhansan (BHS), and Jongno(JN), in order to analyze the spatial and temporal distribution characteristics of ambient VOCs in Seoul.

**Figure 1:**
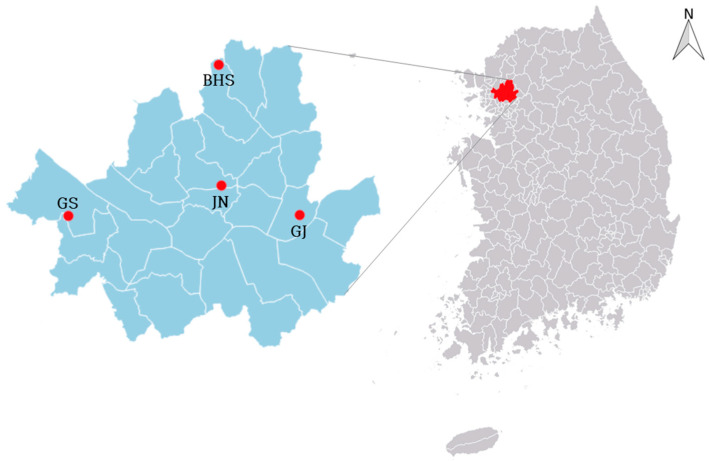
Measurement Sites in This Study.

The four measurement sites selected in this study represent different emission environments within Seoul. The GS site, located in the western part of Seoul, is characterized by complex emission sources influenced by both industrial activities and heavy traffic. The JN site represents a central commercial district with high population density and traffic volume. The GJ site, where residential and commercial areas coexist, exhibits intermediate urban pollution characteristics. In contrast, the BHS site, located in a northern mountainous area, serves as a background site suitable for assessing regional pollution levels and long-range transport influences. Through the selection of these sites, this study aimed to comprehensively investigate the characteristics and spatial variability of VOC emission sources across Seoul. The locations of the measurement sites used in this study are shown in Figure 1.

### 2.2. Measurement and Analytical Methods

Detailed information regarding the measurement and analytical methods for VOCs, as well as quality assurance and quality control (QA/QC) procedures used in this study, is provided in the Supplementary Information.

In this study, to ensure the reliability of the dataset while considering the photochemical loss and uncertainty associated with highly reactive VOCs, only compounds with detection rates greater than 50% among the 56 measured VOC species were selected for analysis. The detection rate was defined as the proportion of measurements with concentrations above the method detection limit (MDL) among the total number of observations [15]. This criterion was applied to minimize uncertainty caused by measurements below MDL and to improve the reliability of subsequent statistical analyses such as correlation analysis and PCA. As a result, 16 VOC species were ultimately selected from the 56 measured compounds, and the detection rates and selection status of all VOC species are presented in Table S3. In addition, the selected compounds included major toxicologically relevant VOCs (e.g., Benzene and aromatic hydrocarbons), important O_3_ precursors (e.g., Ethylene and Propylene), and representative source tracers commonly used in urban source characterization studies. Therefore, despite the exclusion of species with low detection frequencies, the selected VOCs were considered sufficient to represent the major characteristics and source-related behavior of ambient VOCs in Seoul. The selected VOCs included alkanes (Ethane, Propane, Isobutane, n-Butane, Isopentane, n-Pentane, Hexane, and n-Decane), alkenes (Ethylene and Propylene), alkynes (Acetylene), and aromatic compounds (Benzene, Toluene, Ethylbenzene, m/p-Xylene, and o-Xylene). Concentrations below MDL were replaced with one-half of the MDL value prior to statistical analyses. This approach is commonly applied in atmospheric studies to minimize bias associated with non-detect observations while maintaining dataset continuity.

### 2.3. Data Processing

The collected data were analyzed using descriptive statistical methods, including mean and standard deviation, to investigate the spatial distribution and variability of VOC concentrations in Seoul. In addition, diurnal variation characteristics of VOCs were examined based on hourly average concentrations from 00:00 to 23:00. To evaluate the emission characteristics of major sources such as vehicle emissions and solvent usage, the concentration ratio of Toluene to Benzene (Toluene/Benzene ratio, T/B/ratio) was calculated and compared among sites. Furthermore, Pearson correlation analysis was conducted to assess the similarity of VOC compositions and the commonality of emission sources among measurement sites (*p* < 0.05). To evaluate the spatial heterogeneity of VOC distribution characteristics between sites, the Coefficient of Divergence (COD) was calculated. COD values lower than 0.3 were interpreted as indicating similar concentration characteristics between sites, whereas values greater than 0.3 suggested substantial differences in VOC characteristics among sites [16].

Statistical analyses were performed using IBM SPSS Statistics (version 23.0), and data visualization was conducted in Visual Studio Code Version 1.119 using Python (version 3.9.7). To evaluate the statistical significance of spatial and seasonal differences in VOC concentrations, the non-parametric Kruskal–Wallis test was performed using daily mean concentrations because VOC concentration data did not satisfy normality assumptions. Statistical significance was determined at *p* < 0.05.

### 2.4. Principal Component Analysis (PCA)

Principal component analysis (PCA) was performed to identify the major variation characteristics and potential emission sources of VOCs. PCA is a multivariate statistical technique widely used to reduce the dimensionality of datasets by transforming correlated variables into a smaller number of independent components (principal components), thereby facilitating the identification of relationships among pollutants and their common emission sources [6,17].

Prior to conducting PCA, Z-score standardization was applied to eliminate the influence of differences in variable scales. In addition, Varimax rotation was employed to improve the interpretability of the analysis results by enhancing the explanatory power of each component [18]. Principal components were selected based on eigenvalues greater than 1.0, and components were retained until the cumulative explained variance exceeded 70% [19]. The relationships among VOC species were evaluated based on the factor loading values of each principal component, and potential emission sources were interpreted according to the major contributing compounds. In general, compounds with high factor loadings were considered to originate from common emission sources or to share similar chemical characteristics [6].

## 3. Results and Discussion

### 3.1. Concentration of VOCs

**Figure 2:**
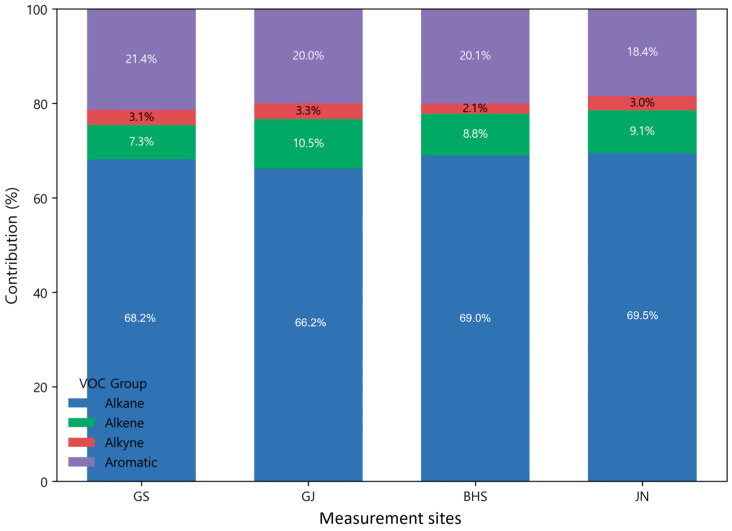
Composition of VOCs in the Measurement Sites.

The mean concentrations, standard deviations, and maximum concentrations of the 16 VOC species measured at the four measurement sites in Seoul are summarized in Table 1, and the compositional profiles for each site are illustrated in Figure 2. Overall, alkanes exhibited the highest concentrations at all sites, followed by aromatic compounds. In contrast, alkenes and alkynes showed relatively lower concentrations. Although the relative composition of VOC groups appeared similar among sites, substantial differences were observed in absolute concentrations (Table 1). This indicates that common VOC group profiles were present across Seoul, whereas the magnitude of VOC concentrations differed according to local emission characteristics and site-specific influences. These results are consistent with previous studies reporting that alkanes are major components of urban atmospheric VOCs originating primarily from fuel evaporation and vehicle emissions [20,21], indicating that the VOC distribution characteristics in Seoul generally follow typical urban atmospheric patterns. Several VOC species exhibited maximum concentrations substantially higher than their long-term mean values, indicating the occurrence of episodic emission events or short-term accumulation episodes. However, investigation of individual high-concentration episodes was beyond the scope of the present study and warrants future analysis.

### 3.2. Spatial and Seasonal Variations in VOC Concentrations

Comparison among the measurement sites showed that VOC concentrations were generally higher at the GS and JN sites. In particular, the major compounds observed at the GS site were Propane (7.25 ppb), n-Butane (7.67 ppb), Ethane (5.09 ppb), and Toluene (4.41 ppb), whereas Ethane (6.59 ppb), Propane (5.78 ppb), and Toluene (3.68 ppb) were dominant at the JN site. These results reflect the characteristics of the GS site, which is affected by both industrial activities and traffic emissions, and the JN site, which represents a heavily trafficked urban area. In contrast, the BHS site exhibited the lowest concentrations for all VOC species, indicating the characteristics of a background area with relatively limited influence from direct anthropogenic emission sources.

**Figure 3:**
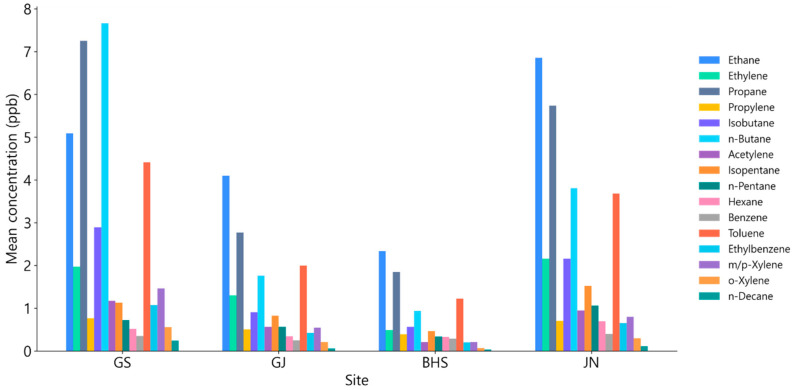
Concentration of VOCs in the Measurement Sites.

**Figure 4:**
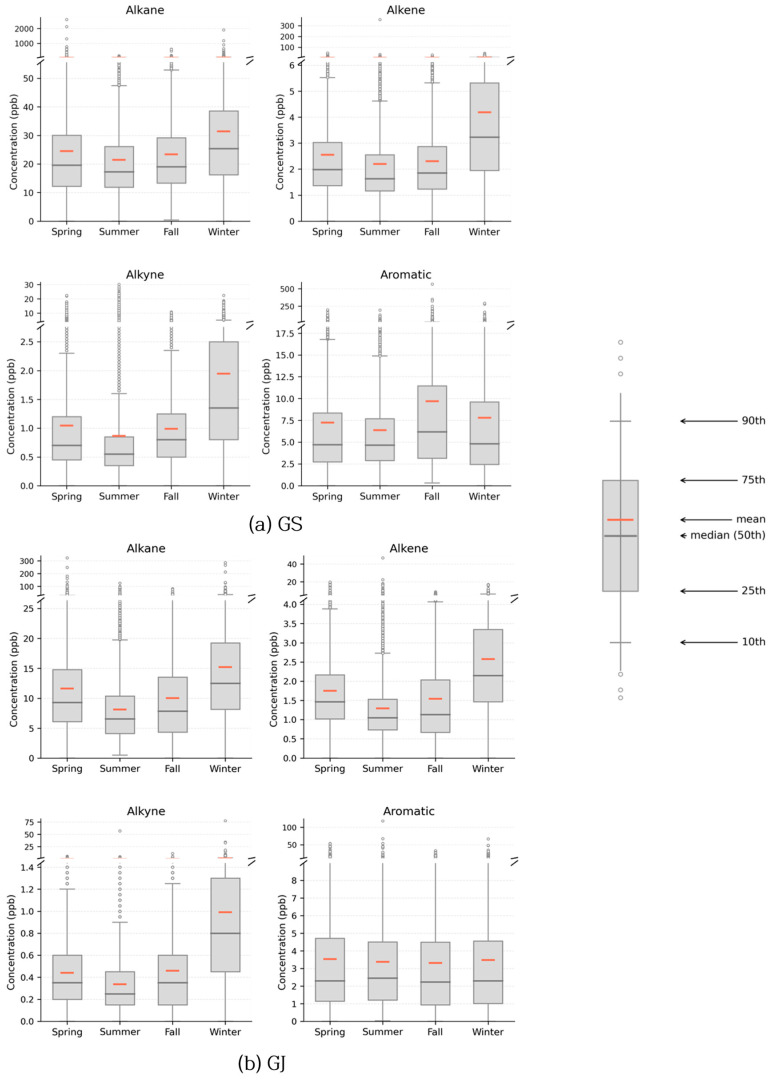

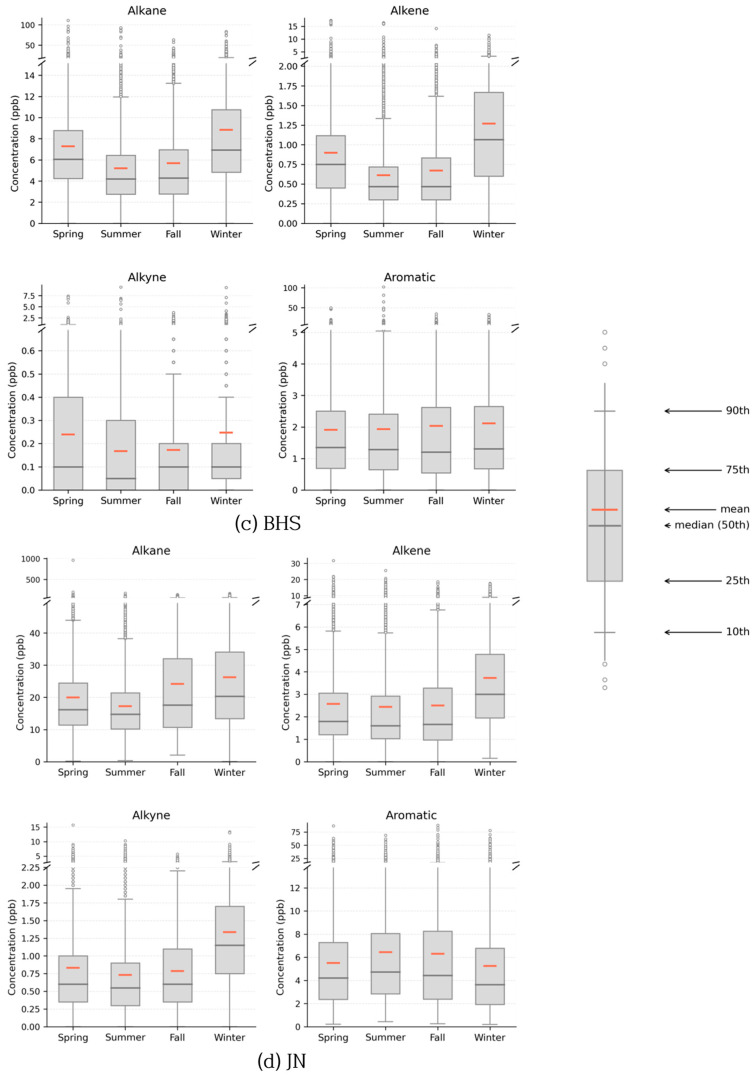
Seasonal Variations in VOCs in the Measurement Sites (**a**,**b**). Seasonal Variations in VOCs in the Measurement Sites (**c**,**d**).

The spatial and seasonal variation characteristics of VOC concentrations at each measurement site are presented in Figure 3 and Figure 4, while the seasonal mean concentrations and standard deviations for each site are summarized in Tables S4–S7. As described earlier, VOC concentrations were generally higher at the GS and JN sites and lower at the BHS site, where Bukhan Mountain is located. Based on these spatial differences, additional analyses were conducted to investigate the seasonal variation characteristics of VOCs at each site.

The observed spatial variability suggests that local emission environments play an important role in determining ambient VOC levels in Seoul. The consistently higher concentrations observed at the GS and JN sites may reflect the combined influence of traffic-related activities, commercial land use, and dense urban development. In contrast, the lower concentrations measured at the BHS site are consistent with its background characteristics and reduced influence of direct anthropogenic emissions. These findings indicate that VOC concentrations in Seoul exhibit substantial spatial heterogeneity despite the relatively compact urban area, highlighting the importance of site-specific emission characteristics [14]. These findings suggest that spatial differences in VOC concentrations are not solely attributable to emission intensity but also reflect the interaction between local emission characteristics and atmospheric dispersion conditions. The clear concentration gradient observed among measurement sites highlights the importance of considering site-specific emission environments when developing VOC management strategies in urban areas. Furthermore, the observed seasonal patterns indicate that both source activities and atmospheric processes contribute to temporal variability in VOC concentration across Seoul.

Mean ∑VOC concentrations differed substantially among the measurement sites, ranging from 9.94 ± 6.21 ppb at BHS to 36.63 ± 37.15 ppb at GS. Intermediate concentrations were observed at GJ (17.14 ± 13.11 ppb) and JN (31.60 ± 21.77 ppb). These results indicate substantial spatial variability in VOC levels across Seoul and are generally consistent with previous urban VOC studies reporting elevated concentrations in traffic-influenced and densely developed urban environments compared with suburban or background locations [14,22]. The observed concentration gradient further supports the importance of local emission characteristics in determining ambient VOC levels. The Kruskal–Wallis test confirmed statistically significant differences in daily mean ∑VOC concentrations among measurement sites (*p* < 0.05), supporting the observed spatial variability of VOC concentrations across Seoul.

Seasonal distribution characteristics of VOCs exhibited distinct patterns depending on the chemical group. Low-molecular-weight alkanes showed the highest concentrations during winter at all measurement sites. In particular, Ethane and Acetylene consistently exhibited elevated wintertime concentrations across all sites, which is likely attributable to enhanced pollutant accumulation caused by lower mixing heights and stagnant atmospheric conditions during winter, combined with increased combustion emissions associated with heating fuel usage [23]. In contrast, aromatic compounds showed site-specific seasonal characteristics. At the GS site, Toluene and m/p-Xylene exhibited the highest concentrations during autumn, whereas at the GJ, BHS, and JN sites, m/p-Xylene and o-Xylene showed relatively higher concentrations during summer. These patterns are considered to result from the combined effects of continuous anthropogenic emissions, such as traffic emissions and solvent use, along with increased evaporative emissions under higher temperature conditions [24].

The pronounced increase in alkane concentrations during winter is likely associated with reduced atmospheric mixing, lower boundary-layer heights, and weaker photochemical removal processes. In contrast, aromatic compounds exhibited greater site-to-site variability than alkanes, suggesting stronger influences from local emission sources such as solvent use, industrial activities, and commercial operations. These results indicate that seasonal VOC variability in Seoul is governed by both meteorological conditions and differences in source characteristics among monitoring environments [25].

Comparison of seasonal variations among the measurement sites revealed that the JN site exhibited the most pronounced increase in VOC concentrations during winter, which was interpreted as the combined effect of intensive urban traffic emissions and seasonal meteorological conditions. The GS site showed seasonal patterns similar to those observed at the JN site; however, it maintained relatively high VOC concentrations throughout the year due to the continuous influence of both industrial and traffic emission sources. In contrast, the BHS site generally exhibited lower VOC concentrations and relatively smaller seasonal variations, reflecting the characteristics of a background area [26]. The interpretation of seasonal variability in this study was based on well-established meteorological characteristics reported in previous studies. However, direct incorporation of meteorological parameters such as temperature, wind speed, and mixing layer height was beyond the scope of the present study and should be considered in future investigations to provide a more comprehensive understanding of VOC variability.

### 3.3. Temporal Characteristics of VOCs

**Figure 5:**
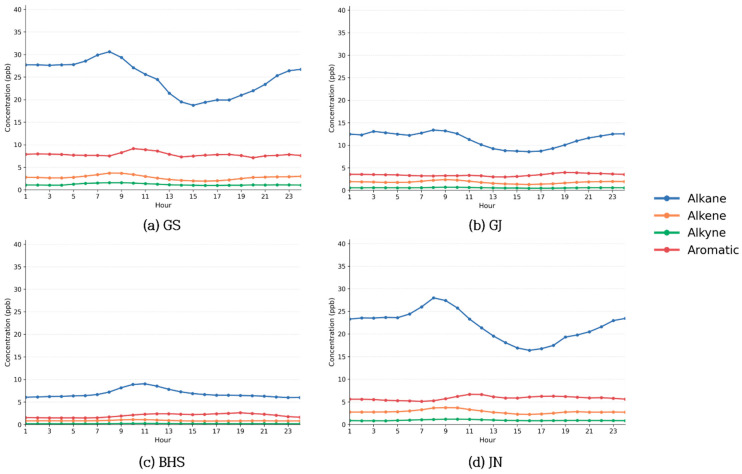
Diurnal Variation in VOC Group Concentrations in the Measurement Sites.

The diurnal variation patterns of VOC groups at each measurement site are presented in Figure 5, while the seasonal diurnal patterns (summer and winter) are shown in Figure S1. The annual diurnal profiles generally exhibited increasing concentrations during the morning and evening periods, with decreasing concentrations during the afternoon. This bimodal pattern is considered to result from increased emissions associated with rush-hour traffic, together with meteorological conditions favorable for pollutant accumulation due to lower mixing heights during the morning and evening periods [27,28]. Aromatic compounds showed relatively elevated or sustained concentrations during daytime hours at the GS and JN sites. This pattern is likely associated with increased solvent usage and fuel evaporation driven by higher daytime temperatures [25].

Distinct seasonal differences were also observed in the diurnal variation patterns. During winter, VOC concentrations were generally higher and exhibited larger fluctuations, which is likely attributable to meteorological conditions favorable for pollutant accumulation, along with increased seasonal emissions such as heating-related combustion. These findings are consistent with the results reported by Kim et al. [29], who observed elevated VOC concentrations under low-temperature conditions in East Asian urban environments. In contrast, VOC concentrations during summer were relatively lower and showed more moderate diurnal variations. This pattern is considered to result from enhanced photochemical reactions under high temperatures and strong solar radiation, leading to rapid VOC consumption, as well as increased atmospheric dispersion during summer conditions [14,30].

### 3.4. Inter-Species Relationships and Source-Related Indicators

**Figure 6:**
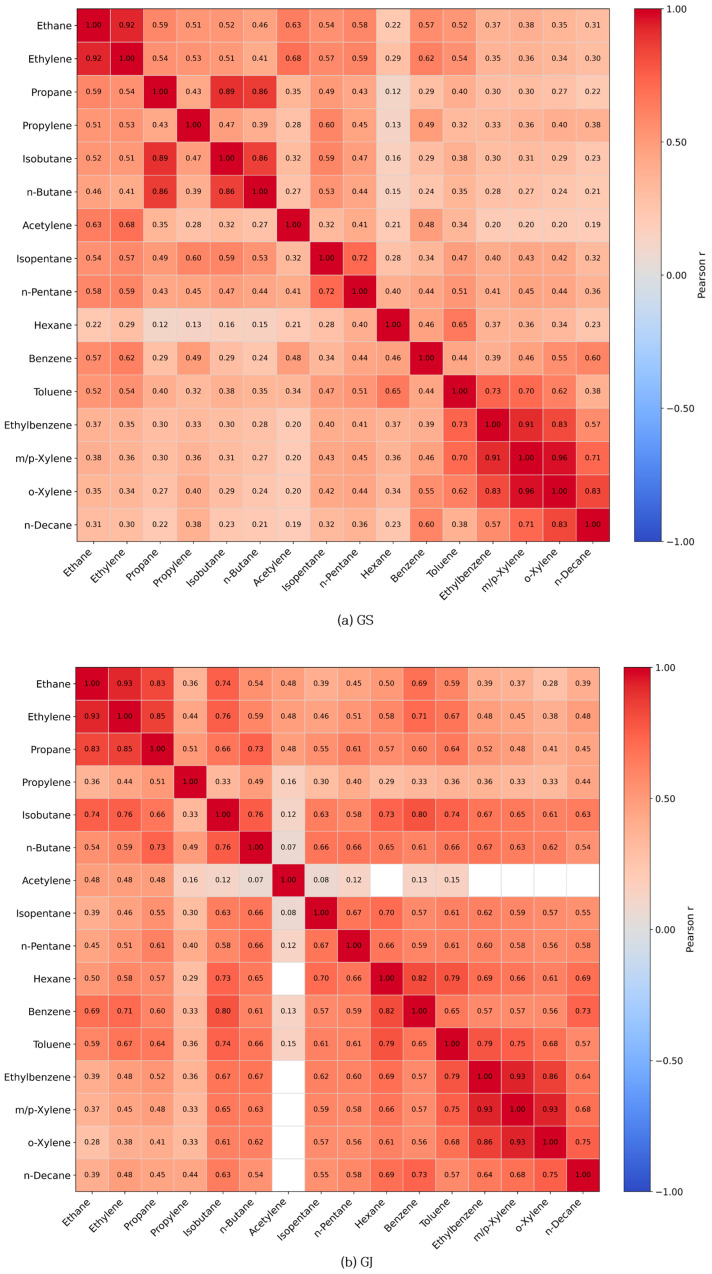

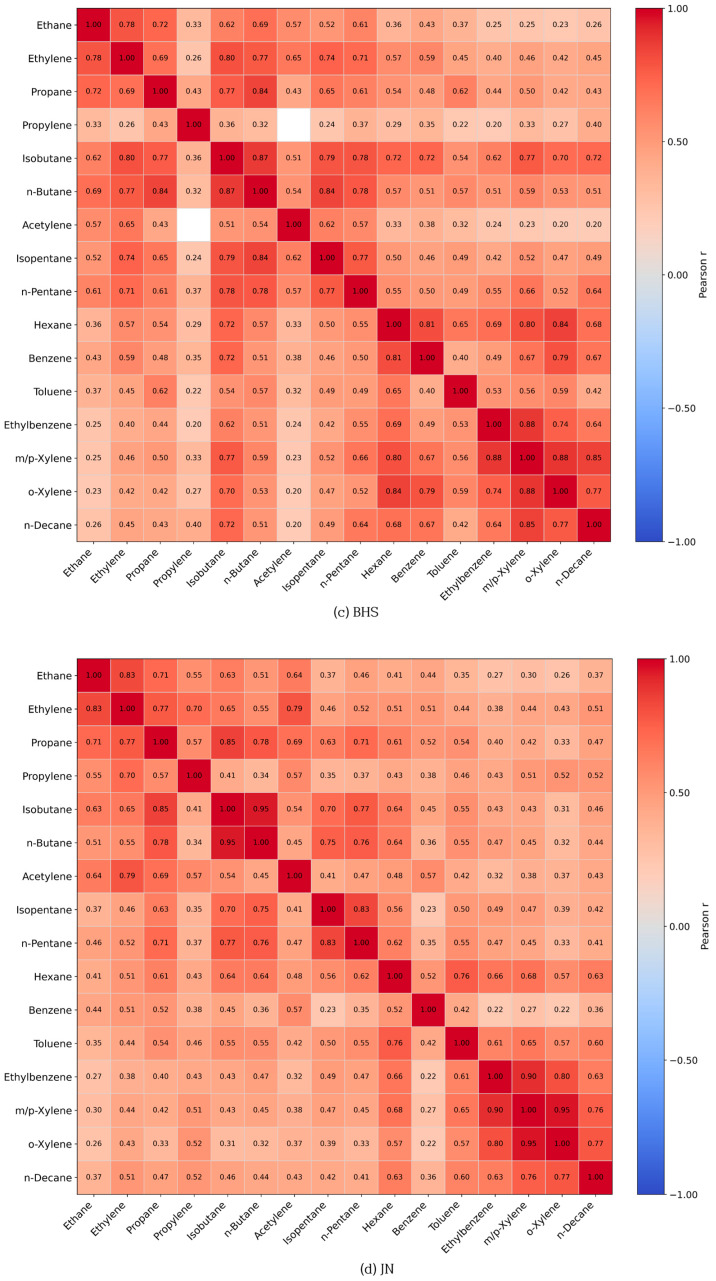
Pearson Correlation Analysis between VOCs in the Measurement Sites (*p* < 0.05, 24 h average).

Pearson correlation analysis was conducted to evaluate the relationships among VOC species at each measurement site, and the results are presented in Figure 6. Overall, positive correlations were observed among most VOC species, with particularly strong correlations identified between compounds belonging to the same chemical group or sharing similar emission sources. Among the alkane species, high correlation coefficients (r > 0.7) were observed at most sites, suggesting the influence of common emission sources, particularly fuel combustion and traffic emissions. In addition, aromatic compounds exhibited very strong correlations (r > 0.8), indicating that these compounds likely originate from similar anthropogenic sources such as solvent use and vehicle emissions [31,32]. In contrast, Acetylene showed relatively weaker correlations with some VOC species. This finding is interpreted as reflecting its distinct emission characteristics, as Acetylene is primarily emitted from incomplete combustion processes and therefore differs from other VOCs in terms of source profiles [33].

Site-specific analysis showed that stronger correlations among VOC species were observed at the GS and JN sites, which are heavily influenced by urban activities and traffic emissions, whereas relatively weaker correlations among certain compounds were identified at the BHS site, characterized as a background area. These results suggest that urban sites are affected by complex mixed emission sources containing various VOC species, while at the background site, the influence of long-range transport and atmospheric dilution may reduce the correlations among VOCs [34].

**Figure 7:**
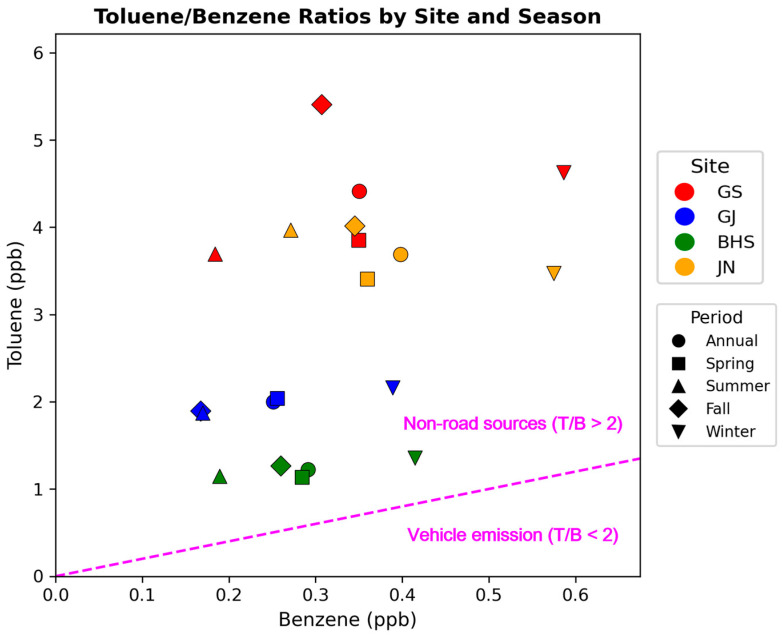
Seasonal Ratio of Toluene/Benzene (T/B) in the Measurement Sites.

The Toluene/Benzene ratio (T/B ratio) is widely used as an indicator for distinguishing major VOC emission sources. In this study, annual and seasonal T/B ratios were calculated for each measurement site and are presented in Figure 7. A T/B ratio lower than 2 is generally interpreted as indicating dominant influence from vehicle emissions, whereas a T/B ratio greater than 2 reflects the influence of non-road emission sources such as solvent use and industrial activities [12]. The T/B ratios calculated in this study were greater than 2 at all measurement sites, with relatively higher values observed at the GS and JN sites. These results suggest that, in addition to vehicle emissions, emissions associated with solvent-related activities such as painting, printing, and cleaning processes substantially contributed to VOC concentrations at these locations.

The T/B ratio tended to increase during summer and autumn, whereas lower values were observed during winter. This pattern is likely attributable to enhanced solvent evaporation and more active photochemical reactions under high-temperature conditions, which promote the emission and formation of aromatic compounds. In contrast, the lower T/B ratio observed during winter is considered to reflect the stronger influence of traffic emissions, which have been identified as a major source of urban VOCs [29]. However, interpretation of the T/B ratio should be made with caution because atmospheric aging, differential photochemical degradation, and meteorological conditions can also influence the observed ratio. Therefore, the T/B ratio was used in this study as a supplementary indicator rather than a definitive source identification tool. Beyond confirming expected relationships among VOC species, the correlation analysis provided supporting evidence for the existence of common source influences across measurement sites and helped identify differences in source mixing characteristics between urban and background environments. These findings also supported the interpretation of subsequent PCA results.

### 3.5. Spatial Similarity and Multivariate Pattern Analysis

**Figure 8:**
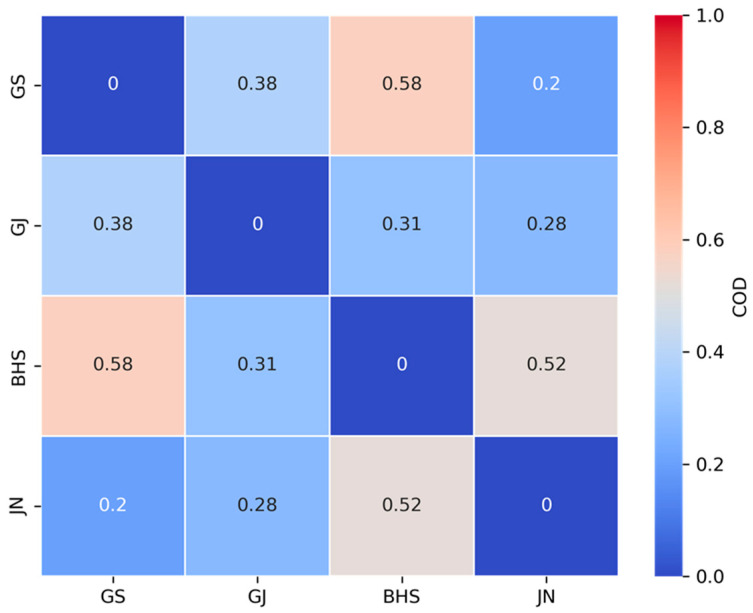
Coefficient of Divergence (COD) between Measurement Sites.

The Coefficient of Divergence (COD) was calculated to evaluate the similarity of VOC distribution characteristics among the measurement sites, and the results are presented in Figure 8. The COD value between the GS and JN sites was the lowest at 0.20, indicating that the VOC compositions at these two sites were highly similar. In contrast, the COD values between GS and BHS and between JN and BHS were 0.58 and 0.52, respectively, demonstrating clear differences in VOC distribution characteristics between urban and background areas. The GJ site exhibited intermediate COD values compared with the other sites (GJ-GS: 0.38, GJ-BHS: 0.31, and GJ-JN: 0.28), suggesting that this site possesses transitional characteristics reflecting both urban and background influences simultaneously [31].

These results suggest that the urban sites, GS and JN, share similar emission source structures, whereas the BHS site exhibits relatively distinct emission characteristics and is more strongly influenced by long-range transport and natural background conditions. This finding is consistent with the study by Park et al. [26], which reported that urban VOC concentrations are predominantly governed by anthropogenic emission sources, while background areas are more strongly affected by atmospheric dispersion and long-range transport processes.

In this study, only the major principal components satisfying the Kaiser [17] criterion (eigenvalue > 1) were selected from the PCA results and are summarized in Table 2, while the score plots and loading plots for each measurement site are presented in Figure S2. At all measurement sites, PC1 was the dominant factor, explaining 47.8–58.7% of the total variance. In PC1, low-molecular-weight alkanes and alkenes, including Ethane, Propane, n-Butane, Isobutane, Ethylene, and Propylene, together with aromatic compounds such as Benzene, Toluene, Ethylbenzene, and Xylene, exhibited high factor loadings simultaneously. These results are consistent with previous studies of urban atmospheric VOCs that reported strong associations between traffic-related activities and major VOC species. Considering that most VOC species showed substantial loadings on PC1, this component likely reflects a mixed urban emission signal strongly influenced by traffic-related activities rather than a single distinct emission source [12,35,36]. Although PCA provides useful information regarding potential source-related patterns, the identified components should be interpreted as indicative rather than definitive source categories. Future studies employing PMF and emission inventory data will be necessary to quantitatively resolve source contributions and further validate the source characteristics suggested by the present analysis.

PC2 explained 12.8–15.4% of the total variance and showed high factor loadings for aromatic compounds such as Ethylbenzene, m/p-Xylene, and o-Xylene. This component may be associated with non-road emission activities, including solvent use, industrial processes, and construction-related emissions, because these sources are commonly linked to elevated aromatic VOC concentrations. According to Askari and Chan [37], buildings and volatile chemical products (VCPs) can make substantial contributions to urban VOC emissions and have been identified as important sources of aromatic compounds.

PC3 observed at the GS, BHS, and JN sites explained approximately 6.5–9.3% of the total variance; however, no specific compounds exhibited dominantly high factor loadings, making it difficult to clearly identify potential source characteristics. This component may reflect the influence of mixed emission environments or secondary atmospheric processes, although definitive interpretation is limited. Similar observations were reported by Alibeknov et al. [38], who noted that certain principal components may contain characteristics of multiple contributing sources, resulting in interpretational ambiguity.

The PCA score plots for each measurement site showed that the GS and JN sites exhibited similar distribution patterns, suggesting the possibility of common source influences at these locations. In contrast, the BHS site displayed a relatively broader distribution pattern, which may indicate a greater influence of regional transport and background atmospheric conditions. These spatial differences were generally consistent with the COD analysis results and suggest differences in VOC distribution characteristics among the measurement sites. In the loading plots, alkanes, alkenes, and aromatic compounds formed clustered patterns in the same direction along PC1, whereas aromatic compounds tended to separate along PC2. These patterns suggest that urban VOC variability in Seoul may be largely influenced by traffic-related activities while also reflecting the contribution of solvent-related and other non-road emission sources. However, PCA identifies covariance structures among VOC species rather than directly quantifying emission sources, and the results should be interpreted as indicative of source-related patterns rather than definitive source attribution. The PCA results presented in this study were based on the complete monitoring dataset to identify dominant long-term source-related patterns. Although seasonal and interannual variability in factor structures may exist, evaluation of the temporal stability of PCA factors was beyond the scope of the present study and warrants further investigation.

From a management perspective, the results suggest that VOC reduction strategies in Seoul should not focus exclusively on traffic emissions. Although traffic-related activities appear to exert a strong influence on overall VOC variability, aromatic compounds associated with solvent use, industrial processes, and other non-road emission activities also contributed to the observed concentration patterns. Therefore, effective VOC management may require integrated control strategies addressing transportation, solvent-related emissions, and industrial activities simultaneously [3].

## 4. Conclusions

This study provides one of the few long-term, multi-site evaluations of VOC characteristics in Seoul using hourly monitoring data collected between 2018 and 2024. By comparing four sites representing distinct emission environments, the study establishes a comprehensive baseline dataset for future source-apportionment and risk-oriented VOC management strategies.

The results showed that ambient VOC concentrations in Seoul exhibited clear spatial differences depending on the emission environment of each site. Higher VOC concentrations were observed at the GS site, which is influenced by both industrial activities and traffic emissions, and at the JN site, characterized by intensive urban traffic. In contrast, the BHS site exhibited the lowest overall concentrations and relatively smaller seasonal variations, reflecting the characteristics of a background area. Seasonal variation patterns of VOCs differed according to chemical groups. Low-molecular-weight alkanes and combustion-related compounds showed elevated concentrations during winter at all sites, which is considered to result from the combined effects of stagnant atmospheric conditions and increased combustion emissions associated with heating during winter. In contrast, aromatic compounds exhibited site-specific seasonal characteristics, with relatively higher concentrations observed during summer or autumn at some sites. These results suggest that, in addition to traffic emissions, solvent use and evaporative emissions also significantly contributed to atmospheric VOC levels.

The diurnal variation patterns commonly exhibited a bimodal distribution, with concentration peaks occurring during morning and evening rush hours, indicating the direct influence of vehicle emissions. In addition, the T/B ratio results suggested that traffic- and combustion-related emissions were the dominant controlling factors, while non-road emission sources such as solvent use and industrial activities, particularly associated with aromatic VOCs, also played important roles. Consistently, the PCA results showed that PC1 represented mixed emission sources related to traffic and combustion processes, whereas PC2 reflected emission characteristics associated with solvent use and industrial activities, mainly characterized by aromatic compounds.

This study confirmed that the distribution characteristics of VOCs in Seoul are influenced by a complex interplay of emission environments, seasonal meteorological conditions, and chemical properties of VOC species. The findings suggest that, in establishing VOC management policies for Seoul, not only traffic emissions but also non-road emission sources such as solvent use and industrial activities should be comprehensively considered. Although health risk assessment was not conducted in the present study, the identified spatial and temporal characteristics of VOCs provide important baseline information for future risk assessment and source-apportionment studies.

## Figures and Tables

**Table 1:** Concentration of VOCs in the Measurement Sites (Unit: ppb).

VOCs	GS			GJ			BHS			JN		
	Mean	S.D. ^a^	Max	Mean	S.D.	Max	Mean	S.D.	Max	Mean	S.D.	Max
Ethane	5.09	4.52	79.05	4.09	3.08	189.85	2.34	1.80	35.70	6.86	6.64	130.85
Ethylene	1.97	1.88	30.70	1.30	1.15	46.00	0.49	0.60	10.00	2.16	2.02	30.15
Propane	7.25	13.93	1435.23	2.77	2.15	88.93	1.85	1.41	31.13	5.74	4.56	336.37
Propylene	0.77	1.96	354.20	0.51	0.40	10.23	0.39	0.41	11.67	0.71	0.55	10.83
Isobutane	2.89	4.02	314.00	0.91	1.02	39.40	0.57	0.59	21.00	2.16	2.08	238.80
n-Butane	7.67	10.30	826.40	1.76	2.10	137.93	0.94	0.98	26.65	3.81	3.65	380.75
Acetylene	1.18	1.53	30.20	0.56	0.82	77.85	0.21	0.34	9.45	0.95	0.84	15.70
Isopentane	1.13	1.47	119.00	0.83	1.72	267.38	0.47	0.54	19.28	1.53	1.32	20.00
n-Pentane	0.72	0.85	35.42	0.57	1.43	257.04	0.34	0.52	29.74	1.06	0.97	59.88
Hexane	0.52	0.68	20.68	0.35	0.60	13.75	0.33	0.51	18.33	0.70	0.67	23.75
Benzene	0.35	0.56	42.05	0.25	0.37	10.77	0.29	0.35	15.97	0.40	0.28	3.70
Toluene	4.41	5.36	357.57	2.00	2.23	34.96	1.22	1.52	29.93	3.68	4.16	79.33
Ethylbenzene	1.08	2.70	202.50	0.43	0.57	32.08	0.20	0.53	68.15	0.65	0.83	52.58
m/p-Xylene	1.46	3.08	118.26	0.54	0.74	54.34	0.21	0.40	27.83	0.80	0.82	30.58
o-Xylene	0.56	1.38	64.49	0.21	0.29	8.24	0.07	0.13	5.39	0.29	0.31	10.84
n-Decane	0.24	1.20	69.54	0.07	0.17	8.83	0.04	0.07	3.30	0.11	0.11	5.46
Alkane	17.91	27.94	2609.04	9.07	9.29	322.64	5.15	5.51	110.65	17.07	17.09	963.04
Alkene	1.92	2.82	356.95	1.45	1.43	4.77	0.66	0.82	17.33	2.23	2.44	31.62
Alkyne	0.83	1.39	30.20	0.45	0.77	77.85	0.16	0.30	9.45	0.74	0.84	15.70
Aromatic	5.63	9.94	567.23	2.74	3.52	118.91	1.50	2.22	102.15	4.53	5.35	88.02
∑VOC	36.48	37.15	2684.53	17.14	13.11	332.38	9.94	7.97	164.48	31.6	21.77	968.48

^a^ S.D.: Standard Deviation.

**Table 2:** PCA Results for VOCs at the Measurement Sites.

Measurement Sites	PC	Eigenvalue	Variance (%)	Cumulative (%)	Major Species (Loading > 0.5)
GS	PC1	7.655	47.815	47.815	Ethane, Ethylene, Propane, Propylene, Isobutane, n-Butane, Acetylene, Isopentane, n-Pentane, Benzene, Toluene, Ethylbenzene, m/p-Xylene, o-Xylene, n-Decane
	PC2	2.458	15.355	63.170	Ethylbenzene, m/p-Xylene, o-Xylene
	PC3	1.470	9.179	72.349	-
	PC4	1.058	6.609	78.958	-
GJ	PC1	9.401	58.728	57.728	Ethane, Ethylene, Propane, Propylene, Isobutane, n-Butane, Isopentane, n-Pentane, Hexane, Benzene, Toluene, Ethylbenzene, m/p-Xylene, o-Xylene, n-Decane
	PC2	2.054	12.830	71.557	-
BHS	PC1	9.309	58.147	58.147	Ethane, Ethylene, Propane, Isobutane, n-Butane, Acetylene, Isopentane, n-Pentane, Hexane, Benzene, Toluene, Ethylbenzene, m/p-Xylene, o-Xylene, n-Decane
	PC2	2.157	13.472	71.619	o-Xylene
	PC3	1.040	6.498	78.117	-
JN	PC1	8.882	55.481	55.481	Ethane, Ethylene, Propane, Propylene, Isobutane, n-Butane, Acetylene, Isopentane, n-Pentane, Hexane, Benzene, Toluene, Ethylbenzene, m/p-Xylene, o-Xylene, n-Decane
	PC2	2.156	13.469	68.950	Ethylbenzene, m/p-Xylene, o-Xylene
	PC3	1.482	9.256	78.205	-

## Data Availability

Restrictions apply to the availability of these data. Data were obtained from SIHE and are available with the permission of SIHE.

## Supplementary Materials

The following supporting information can be downloaded at: https://www.mdpi.com/article/10.3390/toxics14070554/s1, Figure S1: Seasonal Diurnal Variation in VOC Group Concentrations (Summer and Winter) by the Measurement Sites; Figure S2: PCA Score Plot and Loading Plot Results of VOCs at the Measurement Sites (a–b). PCA Score Plot and Loading Plot Results of VOCs at the Measurement Sites (c–d); Table S1: Measurement and Cut-off Ranges of Valid Observation; Table S2: Method Detection Limits for VOCs by Measurement Sites (Unit: ppb); Table S3: Detection Frequency of 56 Types of VOCs by Measurement Sites; Table S4: Seasonal Concentrations of VOCs in GS (Unit: ppb); Table S5: Seasonal Concentrations of VOCs in GJ (Unit: ppb); Table S6: Seasonal Concentrations of VOCs in BHS (Unit: ppb); Table S7: Seasonal Concentrations of VOCs in JN (Unit: ppb) [39,40].
