# Supplementary material for: Spatial and Temporal Distribution Characteristics of VOCs in Seoul Ambient Air and Identification of Potential Pollution Sources Using Principal Component Analysis
Source: Toxics. 2026 Jun 25;14(7):554. doi: 10.3390/toxics14070554 (PMC13418157; doi:10.3390/toxics14070554)
Supplement: Supplementary file 1 [file toxics-14-00554-s001.zip › toxics-4360698-supplementary.pdf]

toxics

1. Measurement and Analysis of VOCs

Volatile organic compounds (VOCs) were measured using the Photochemical Assessment Monitoring Stations (PAMS) operated by the Seoul Metropolitan Government Research Institute of Health and Environment. The monitoring was conducted in accordance with Article 3 (Continuous Monitoring) and Article 11 (Establishment of Comprehensive Air Quality Improvement Plans) of the Clean Air Conservation Act, as well as the Guidelines for Installation and Operation of Air Pollution Monitoring Networks [39,40].

The VOCs measured at the PAMS sites were analyzed in real time according to the "Automatic Measurement Method for Ozone Precursors in Ambient Air (ES 01805.1)" specified in the Korean Standard Methods for Air Pollution [39]. Samples were collected every hour from 10 to 50 minutes past the hour for a total of 40 minutes using a miniature pump at a flow rate of 15 mL/min, resulting in a total sampling volume of approximately 600 mL. Initially, 56 VOC species were targeted for analysis and quantified using an automated Gas Chromatography/Flame Ionization Detector (GC/FID) system. The collected samples were first dried in a Nafion dryer to remove moisture and subsequently subjected to thermal desorption using an automated thermal desorption system. The desorbed compounds were then separated through two columns within the GC system, and the peaks of the separated compounds were repeatedly measured using the FID detector (Table S1).

Table S1. Measurement and Cut-off Ranges of Valid Observation.

Item	Standard	Test Result	Method
System blank analysis	The sum of total peak < 20ppbv VOCs conc. per each column < 10ppbv VOCs conc.	PLOT: 0.0013	Use humidified N_2 gas
		BP-1: 0.0028	
Multiple points cal. linearity	$R^2 \geq 0.995$	PLOT: 0.9965	1,5,10ppbv as propane, benzene, RT < ±0.5min
		BP-1: 0.9999	
MDL (Method Detection Limit) test	< 2ppbv as propane, benzene	Propane: 0.39	1ppbv 7 measurements in a row
		Benzene: 0.49	
Precision test	Within ±20% RPD	Propane: 1.31	5ppbv 2 measurements
		Benzene: 0.83	
Resolution	56 target components	Normal separation	-

2. Quality Assurance/Quality Control (QA/QC)

Annual quality assurance and quality control (QA/QC) procedures were conducted to ensure the reliability of the analytical results. The evaluated QA/QC parameters included the method detection limit (MDL), relative standard deviation (RSD), and the coefficient of determination (R^2) of the calibration curve. The MDL was calculated by multiplying the standard deviation of seven repeated measurements by 3.14. The RSD(%) was calculated by dividing the standard deviation of repeated measurements of standard materials by the mean value and multiplying by 100; in this study, RSD values were determined based on four repeated measurements. In addition, calibration curves were established using four standard materials within the quantification range, and the linearity of the calibration curves was evaluated using the coefficient of determination (R^2). Calibration curves with R^2 values lower than 0.99 were reconstructed. The QA/QC results obtained in this study demonstrated that all criteria for blank test, R^2, MDL, and precision were satisfied, confirming the reliability of the VOC analytical data (Table S2). The MDLs of the VOC species ranged from 0.013 ppbv for n-Dodecane to 0.119 ppbv for Acetylene, indicating sufficient analytical sensitivity for ambient VOC measurements.

Table S2. Method Detection Limits for VOCs by Measurement Sites (Unit: ppb).

VOCs	Measurement sites			
	GS	GJ	BHS	JN
Ethane	0.0839	0.0766	0.0766	0.0839
Ethylene	0.1083	0.0593	0.0839	0.0839
Propane	0.0996	0.0559	0.0722	0.0396
Propylene	0.0511	0.0559	0.0824	0.0722
i-Butane	0.0383	0.0383	0.0747	0.0747
n-Butane	0.0297	0.0706	0.0890	0.0618
Acetylene	0.1187	0.0593	0.0839	0.0906
trans-2-Butene	0.0420	0.0785	0.0873	0.0641
1-Butene	0.0420	0.0839	0.0706	0.0747
cis-2-Butene	0.0593	0.0453	0.0641	0.0839
Cyclopentane	0.0363	0.0699	0.0565	0.0877
i-Pentane	0.0433	0.0565	0.0597	0.0867
n-Pentane	0.0433	0.0811	0.0867	0.0763
trans-2-Pentene	0.0306	0.0712	0.0919	0.0949
1-Pentene	0.0306	0.0613	0.0867	0.0888
cis-2-Pentene	0.0494	0.0565	0.0494	0.0949
2,2-Dimethylbutane	0.0198	0.0582	0.0722	0.0791
2,3-Dimethylbutane	0.0198	0.0656	0.0704	0.0396
2-Methylpentane	0.0198	0.0471	0.0656	0.0676
3-Methylpentane	0.0427	0.0511	0.0906	0.0899
Isoprene	0.0336	0.0306	0.0867	0.0628
1-Hexane	0.0396	0.0255	0.0906	0.0666
Hexane	0.0511	0.0766	0.0471	0.0899

Methylcyclopentane	0.0847	0.0666	0.0942	0.0783
2,4-Dimethylpentane	0.0876	0.0438	0.0480	0.0499
Benzene	0.0302	0.0361	0.0656	0.0498
Cyclohexane	0.0731	0.0302	0.0839	0.0892
2-Methylhexane	0.0719	0.0404	0.0404	0.0427
2,3-Dimethylpentane	0.0706	0.0404	0.0438	0.0427
3-Methylhexane	0.0657	0.0545	0.0366	0.0848
2,2,4-Trimethylpentane	0.0902	0.0383	0.0499	0.0717
n-Heptane	0.0240	0.0219	0.0619	0.0562
Methylcyclohexane	0.0819	0.0449	0.0571	0.0771
2,3,4-Trimethylpentane	0.0373	0.0353	0.0353	0.0641
Toluene	0.0427	0.0892	0.0765	0.0771
2-Methylheptane	0.0420	0.0271	0.0373	0.0742
3-Methylheptane	0.0383	0.0420	0.0383	0.0873
n-Octane	0.0445	0.0271	0.0437	0.0926
Ethylbenzene	0.0437	0.0192	0.0210	0.0869
m,p-Xylene	0.0383	0.0618	0.0445	0.0752
Styrene	0.0542	0.0210	0.0600	0.0799
o-Xylene	0.0353	0.0383	0.0210	0.0747
n-Nonane	0.0444	0.0186	0.0241	0.0694
i-Propylbenzene	0.0241	0.0444	0.0264	0.0851
n-Propylbenzene	0.0332	0.0403	0.0264	0.0865
m-Ethyltoluene	0.0275	0.0241	0.0424	0.0698
p-Ethyltoluene	0.0469	0.0275	0.0570	0.0698
1,3,5-Trimethylbenzene	0.0264	0.0264	0.0285	0.0898
o-Ethyltoluene	0.0403	0.0275	0.0332	0.0659
1,2,4-Trimethylbenzene	0.0201	0.0522	0.0186	0.0960
n-Decane	0.0256	0.0433	0.0237	0.0744
1,2,3-Trimethylbenzene	0.0373	0.0373	0.0314	0.0632
m-Diethylbenzene	0.0336	0.0620	0.0460	0.0601
p-Diethylbenzene	0.0247	0.0597	0.0283	0.0613
n-Undecane	0.0347	0.0363	0.0165	0.0930
n-Dodecane	0.0128	0.0255	0.0249	0.0827

Table S3. Detection Frequency of 56 Types of VOCs by Measurement Sites.

VOCs	Group	Measurement Sites							
		GS		GJ		BHS		JN	
		N	Detection Frequency (%)	N	Detection Frequency (%)	N	Detection Frequency (%)	N	Detection Frequency (%)
Ethane	Alkane	38,416	99.96	43,788	99.99	41,067	99.90	42,572	99.84
Ethylene	Alkene	38,416	99.24	43,794	99.17	41,067	86.91	42,572	98.86
Propane	Alkane	38,416	99.86	43,773	98.57	41,068	99.12	42,572	99.88
Propylene	Alkene	38,416	91.85	43,793	96.85	41,067	90.23	42,572	97.18
Isobutane	Alkane	38,416	99.17	43,776	99.50	41,067	97.66	42,572	99.63
n-Butane	Alkane	38,416	98.08	43,776	97.87	41,067	93.85	42,572	98.83
Acetylene	Alkyne	38,416	93.49	43,794	85.21	41,067	58.47	42,572	91.85
tans-2-Butene	Alkene	38,416	29.55	43,794	28.69	41,068	12.37	42,572	46.25
1-Butene	Alkene	38,416	64.31	43,794	37.51	41,068	48.88	42,572	77.59
cis-2-Butene	Alkene	38,416	38.17	43,794	50.80	41,068	28.41	42,572	43.37
Cyclopentane	Alkane	38,416	81.05	43,794	55.61	41,068	43.23	42,572	79.78
Isopentane	Alkane	38,416	94.83	43,794	93.70	41,068	86.80	42,572	93.93
n-Pentane	Alkane	38,413	96.63	43,793	90.13	41,068	82.15	42,572	98.93
trans-2-Pentene	Alkene	38,415	29.48	43,794	17.43	41,068	2.81	42,572	38.49
1-Pentene	Alkene	38,416	30.63	43,794	9.71	41,068	3.20	42,572	28.21
cis-2-Pentene	Alkene	38,416	12.85	43,794	8.16	41,068	11.07	42,572	15.50
2,2-Dimethylbutane	Alkane	38,416	39.37	43,794	21.03	41,068	8.72	42,572	39.52

2,3-Dimethylbutane	Alkane	38,383	47.30	43,794	31.81	41,067	13.04	42,572	60.91
2-Methylpentane	Alkane	38,385	78.82	43,794	82.21	41,068	45.40	42,572	80.52
3-Methylpentane	Alkane	38,385	62.85	43,794	47.21	41,068	18.39	42,572	64.95
Isoprene	Alkene	38,385	63.38	43,794	47.68	41,068	30.56	42,572	50.83
1-Hexane	Alkene	38,435	19.95	43,794	26.88	41,068	5.73	42,572	18.61
Hexane	Alkane	39,298	82.03	43,728	64.78	41,094	77.87	42,571	97.26
Methylcyclopentane	Alkane	39,298	59.21	43,728	46.49	41,094	23.93	42,570	74.69
2,4-Dimethylpentane	Alkane	39,298	2.69	43,728	0.30	41,094	2.71	42,570	5.08
Benzene	Aromatic	39,298	78.69	43,727	70.29	41,093	87.27	42,477	91.41
Cyclohexane	Alkane	39,298	35.50	43,728	30.28	41,094	11.47	42,570	36.74
2-Methylhexane	Alkane	39,298	25.34	43,728	23.00	41,094	8.11	42,570	37.96
2,3-Dimethylpentane	Alkane	39,298	4.46	43,728	3.40	41,094	0.96	42,570	12.09
3-Methylhexane	Alkane	39,298	35.01	43,728	31.30	41,094	15.10	42,570	47.21
2,2,4-Trimethylpentane	Alkane	39,298	22.09	43,728	20.60	41,094	5.13	42,570	31.38
n-Heptane	Alkane	39,298	47.32	43,728	39.98	41,094	15.16	42,570	54.89
Methylcyclohexane	Alkane	39,298	47.52	43,728	38.44	41,094	15.61	42,570	52.40
2,3,4-Trimethylpentane	Alkane	39,298	8.88	43,728	14.67	41,094	1.03	42,570	15.04
Toluene	Aromatic	39,298	99.96	43,728	98.63	41,094	96.89	42,570	100.00
2-Methylheptane	Alkane	39,298	13.03	43,728	14.15	41,094	2.22	42,570	7.15
3-Methylheptane	Alkane	39,298	19.30	43,728	10.54	41,094	0.84	42,570	6.64
n-Octane	Alkane	39,298	43.06	43,728	39.84	41,094	15.05	42,570	23.68
Ethylbenzene	Aromatic	39,042	98.44	43,728	96.10	41,094	81.08	42,570	96.08
m,p-Xylene	Aromatic	39,042	98.86	43,728	93.49	41,094	71.08	42,570	96.20
Styrene	Aromatic	39,298	53.92	43,728	40.14	41,094	4.32	42,570	11.76

o-Xylene	Aromatic	39,042	95.93	43,728	85.15	41,094	60.02	42,570	89.82
n-Nonane	Alkane	39,298	51.28	43,728	54.95	41,094	16.96	42,570	34.91
Isopropylbenzene	Aromatic	39,298	12.04	43,728	1.25	41,094	0.50	42,570	2.92
n-Propylbenzene	Aromatic	39,298	14.86	43,728	5.12	41,094	4.28	42,570	2.36
m-Ethyltoluene	Aromatic	39,298	63.04	43,728	40.92	41,094	16.56	42,570	20.18
p-Ethyltoluene	Aromatic	39,042	19.30	43,728	15.34	41,094	0.95	42,570	7.55
1,3,5-Trimethylbenzene	Aromatic	39,298	35.83	43,728	24.22	41,094	4.11	42,570	6.27
o-Ethyltoluene	Aromatic	39,042	23.03	43,728	19.58	41,094	0.95	42,570	6.14
1,2,4-Trimethylbenzene	Aromatic	39,298	86.34	43,728	41.58	41,094	29.21	42,570	34.30
n-Decane	Alkane	39,298	92.14	43,728	51.85	41,094	59.09	42,570	62.52
1,2,3-Trimethylbenzene	Aromatic	39,298	67.14	43,728	41.57	41,094	38.72	42,570	17.52
m-Diethylbenzene	Aromatic	39,298	18.22	43,728	1.55	41,094	0.79	42,570	8.94
p-Diethylbenzene	Aromatic	39,298	25.71	43,728	2.62	41,094	1.02	42,570	2.92
n-Undecane	Alkane	39,298	68.54	43,728	27.76	41,094	23.49	42,570	9.62
n-Dodecane	Alkane	39,298	74.52	43,090	27.47	41,094	7.24	42,570	7.64

Table S4. Seasonal Concentrations of VOCs in GS (Unit: ppb).

VOCs	Spring		Summer		Fall		Winter	
	Mean	S.D.[a]	Mean	S.D.	Mean	S.D.	Mean	S.D.
Ethane	5.12	3.77	3.06	2.21	4.35	3.64	8.27	6.34
Ethylene	1.71	1.61	1.38	1.16	1.74	1.37	3.31	2.68
Propane	7.59	19.94	6.14	4.63	6.18	6.24	9.48	17.58
Propylene	0.84	0.92	0.82	3.88	0.57	0.35	0.88	0.93
Isobutane	2.78	5.55	3.04	2.6	2.65	2.44	3.22	4.49
n-Butane	8.06	14.98	6.76	6.07	7.44	5.74	8.44	10.9
Acetylene	1.05	1.29	0.86	1.91	0.99	0.81	1.95	1.86
Isopentane	1.01	1.65	1.24	1.72	1.17	1.08	1.13	1.37
n-Pentane	0.75	0.97	0.61	0.59	0.72	0.67	0.81	1.09
Hexane	0.46	0.63	0.49	0.58	0.63	0.74	0.46	0.73
Benzene	0.35	0.53	0.18	0.68	0.31	0.31	0.59	0.64
Toluene	3.85	4.12	3.7	3.59	5.41	7.27	4.63	5.21
Ethylbenzene	1.01	1.81	0.83	1.55	1.51	4.23	4.63	5.21
m/p-Xylene	1.45	2.95	1.18	1.9	1.89	3.73	1.22	3.27
o-Xylene	0.57	1.36	0.48	1.01	0.67	1.35	0.48	1.71
n-Decane	0.25	1.04	0.14	0.63	0.24	1.20	0.33	1.74

[a]S.D.: Standard Deviation

Table S5. Seasonal Concentrations of VOCs in GJ (Unit: ppb).

VOCs	Spring		Summer		Fall		Winter	
	Mean	S.D.	Mean	S.D.	Mean	S.D.	Mean	S.D.
Ethane	4.32	2.94	2.22	1.33	3.22	2.31	6.33	3.47
Ethylene	1.20	0.98	0.84	0.90	1.04	0.91	2.05	1.31
Propane	2.67	2.06	1.96	1.29	2.49	1.97	3.90	2.52
Propylene	0.55	0.38	0.45	0.40	0.50	0.40	0.53	0.40
Isobutane	0.96	1.16	0.69	0.73	0.82	0.73	1.13	1.23
n-Butane	1.74	2.40	1.47	1.74	2.00	2.01	1.89	2.12
Acetylene	0.44	0.42	0.34	0.64	0.46	0.46	0.99	1.25
Isopentane	0.88	1.34	0.85	1.14	0.71	0.84	0.84	2.74
n-Pentane	0.61	0.82	0.51	0.71	0.51	0.60	0.62	2.50
Hexane	0.38	0.63	0.34	0.60	0.23	0.33	0.42	0.70
Benzene	0.26	0.38	0.17	0.40	0.17	0.18	0.39	0.41
Toluene	2.04	2.21	1.87	1.84	1.90	2.18	2.16	2.58
Ethylbenzene	0.45	0.59	0.47	0.62	0.46	0.6	0.35	0.47
m/p-Xylene	0.58	0.81	0.60	0.93	0.58	0.58	0.43	0.54
o-Xylene	0.21	0.30	0.27	0.33	0.21	0.21	0.15	0.26
n-Decane	0.07	0.16	0.06	0.19	0.06	0.06	0.07	0.22

Table S6. Seasonal Concentrations of VOCs in BHS (Unit: ppb).

VOCs	Spring		Summer		Fall		Winter	
	Mean	S.D.	Mean	S.D.	Mean	S.D.	Mean	S.D.
Ethane	2.82	1.68	1.28	0.86	1.60	1.15	3.35	2.21
Ethylene	0.50	0.58	0.37	0.53	0.33	0.45	0.73	0.72
Propane	1.81	1.15	1.41	1.12	1.68	1.38	2.43	1.71
Propylene	0.40	0.43	0.25	0.38	0.35	0.30	0.54	0.44
Isobutane	0.57	0.67	0.48	0.62	0.52	0.49	0.67	0.54
n-Butane	0.93	0.95	0.85	0.98	0.83	0.99	1.13	0.97
Acetylene	0.24	0.35	0.17	0.29	0.17	0.29	0.25	0.38
Isopentane	0.46	0.62	0.51	0.58	0.40	0.45	0.51	0.47
n-Pentane	0.35	0.65	0.31	0.63	0.29	0.32	0.39	0.38
Hexane	0.32	0.49	0.33	0.62	0.34	0.48	0.36	0.45
Benzene	0.28	0.36	0.19	0.46	0.26	0.22	0.42	0.28
Toluene	1.13	1.23	1.15	1.34	1.27	1.72	1.35	1.76
Ethylbenzene	0.20	0.31	0.26	1.02	0.22	0.29	0.14	0.22
m/p-Xylene	0.22	0.39	0.25	0.60	0.22	0.32	0.16	0.24
o-Xylene	0.07	0.13	0.08	0.18	0.07	0.10	0.05	0.08
n-Decane	0.04	0.08	0.04	0.10	0.04	0.04	0.04	0.04

Table S7. Seasonal Concentrations of VOCs in JN (Unit: ppb).

VOCs	Spring		Summer		Fall		Winter	
	Mean	S.D.	Mean	S.D.	Mean	S.D.	Mean	S.D.
Ethane	6.68	6.40	4.36	4.94	6.41	6.67	9.48	7.14
Ethylene	1.86	1.92	1.72	1.81	1.93	1.91	2.96	2.12
Propane	5.24	4.74	4.42	3.42	5.97	4.77	7.14	4.72
Propylene	0.71	0.58	0.73	0.71	0.57	0.41	0.77	0.41
Isobutane	1.88	2.71	1.78	1.20	2.65	2.25	2.40	1.83
n-Butane	3.08	4.33	3.32	2.15	5.21	4.49	3.90	3.06
Acetylene	0.83	0.86	0.73	0.78	0.79	0.62	1.34	0.89
Isopentane	1.35	1.10	1.61	1.41	1.84	1.56	1.39	1.19
n-Pentane	0.99	0.72	0.97	0.98	1.19	1.02	1.11	1.08
Hexane	0.63	0.55	0.69	0.57	0.76	0.78	0.72	0.74
Benzene	0.36	0.25	0.27	0.26	0.35	0.24	0.58	0.27
Toluene	3.40	3.42	3.94	4.13	4.02	4.84	0.58	0.27
Ethylbenzene	0.69	1.13	0.79	0.79	0.77	0.80	3.47	4.23
m/p-Xylene	0.79	0.89	1.02	0.99	0.88	0.69	0.57	0.56
o-Xylene	0.27	0.27	0.43	0.46	0.30	0.22	0.20	0.18
n-Decane	0.10	0.08	0.13	0.13	0.12	0.15	0.10	0.08

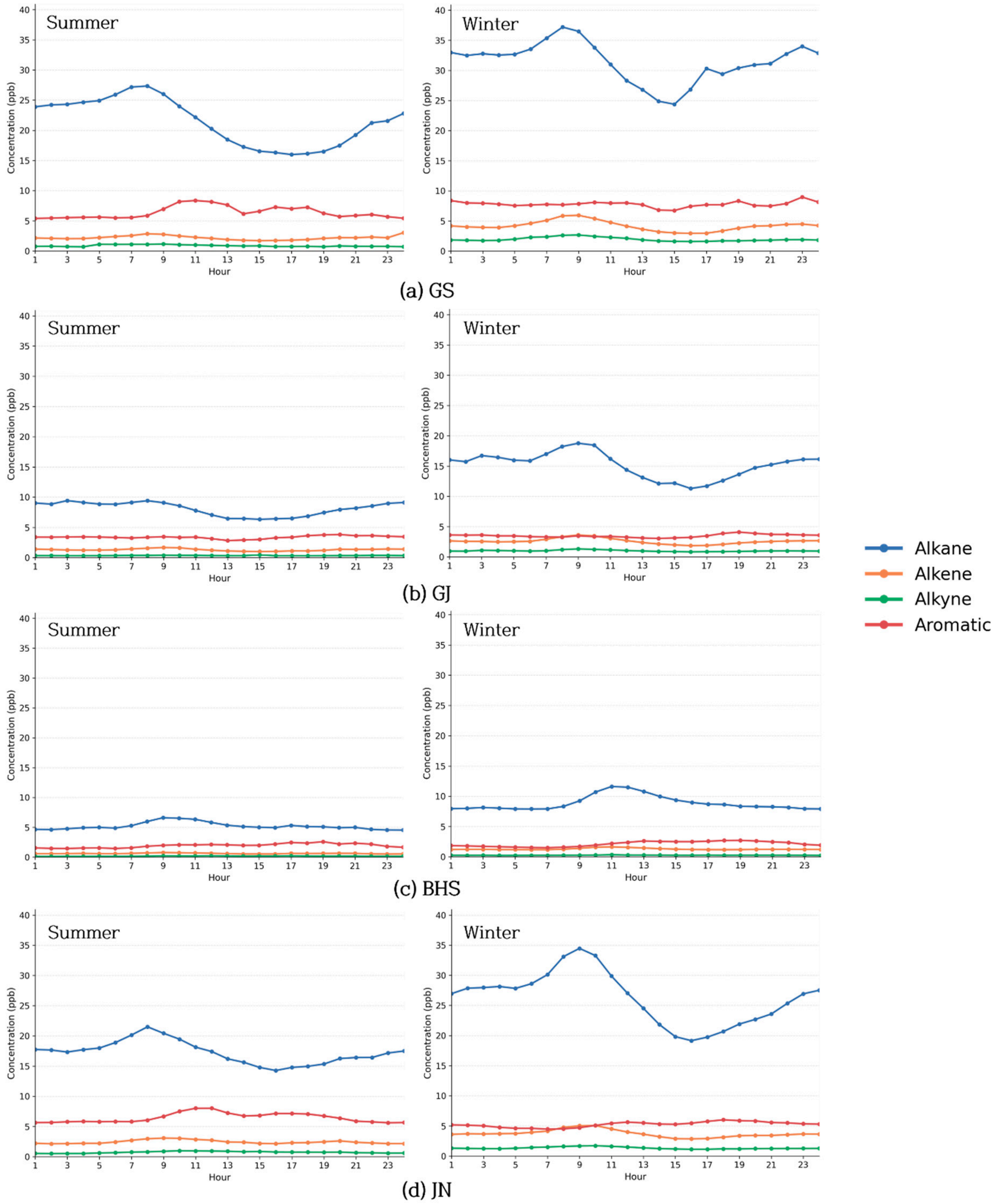

Figure S1. Seasonal Diurnal Variation in VOC Group Concentrations (Summer and Winter) by the Measurement Sites.

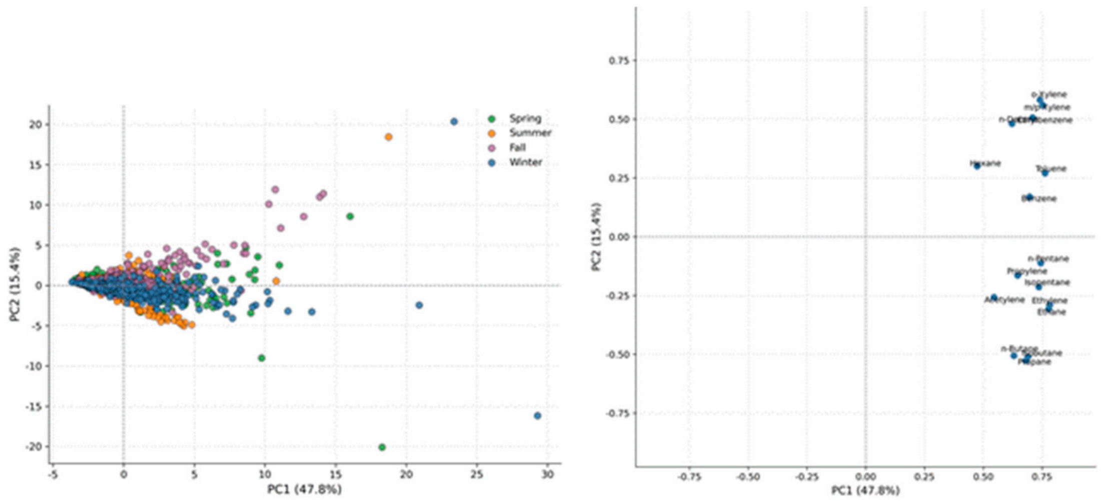
Spring
Summer
Fall
Winter
PC1 (47.8%)
PC2 (15.4%)
o-Xylene
Toluene
Benzene
Isopentane
Ethylene
Propane

(a) GS

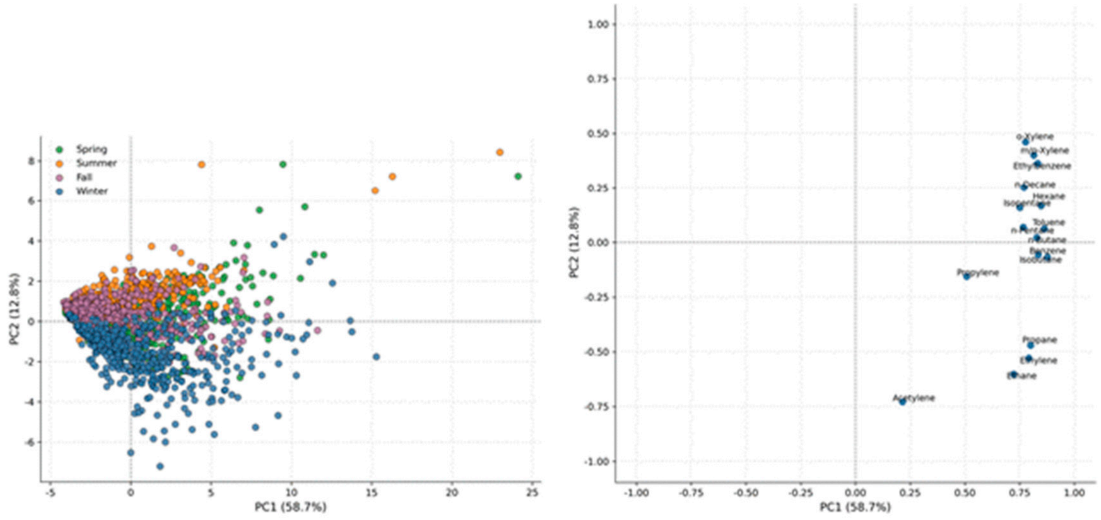
Spring
Summer
Fall
Winter
PC1 (58.7%)
PC2 (12.8%)
o-Xylene
n-Decane
Hexane
Toluene
Propylene
Propane
Ethylene
Ethane
Acetylene

(b) GJ

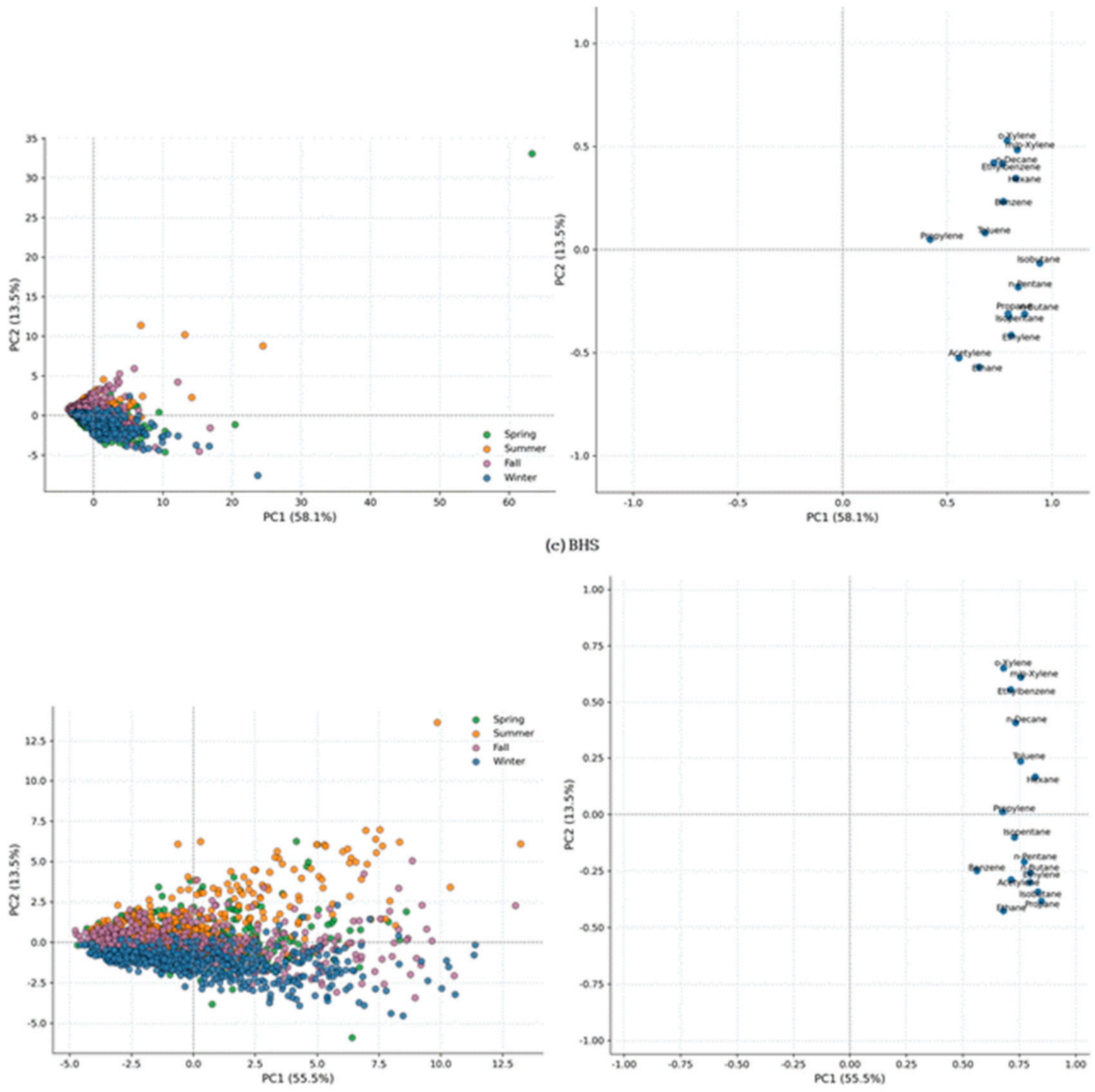

(c) BHS

(d) JN

Figure S2. PCA Score Plot and Loading Plot Results of VOCs at the Measurement Sites (a-b). PCA Score Plot and Loading Plot Results of VOCs at the Measurement Sites (c-d).
